# Supplementary figures and images for: Disintegration half-life of biodegradable plastic films on different marine beach sediments
Source: PeerJ. 2021 Aug 10;9:e11981. doi: 10.7717/peerj.11981 (PMC8362673; doi:10.7717/peerj.11981)

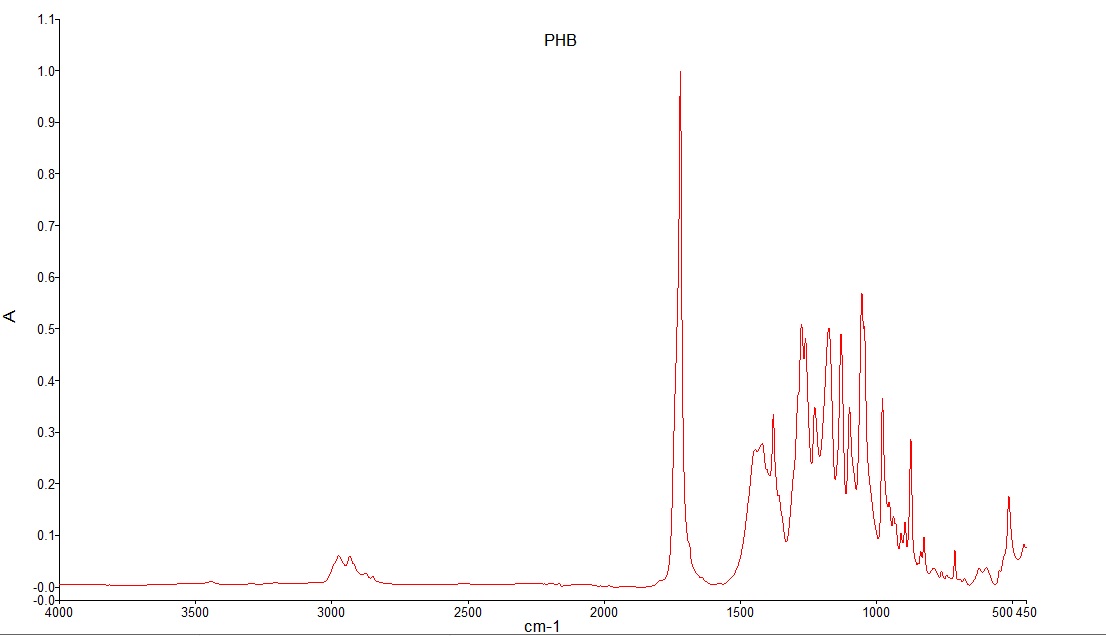

Supplement: Supplemental Information 1 [file peerj-09-11981-s001.jpg]

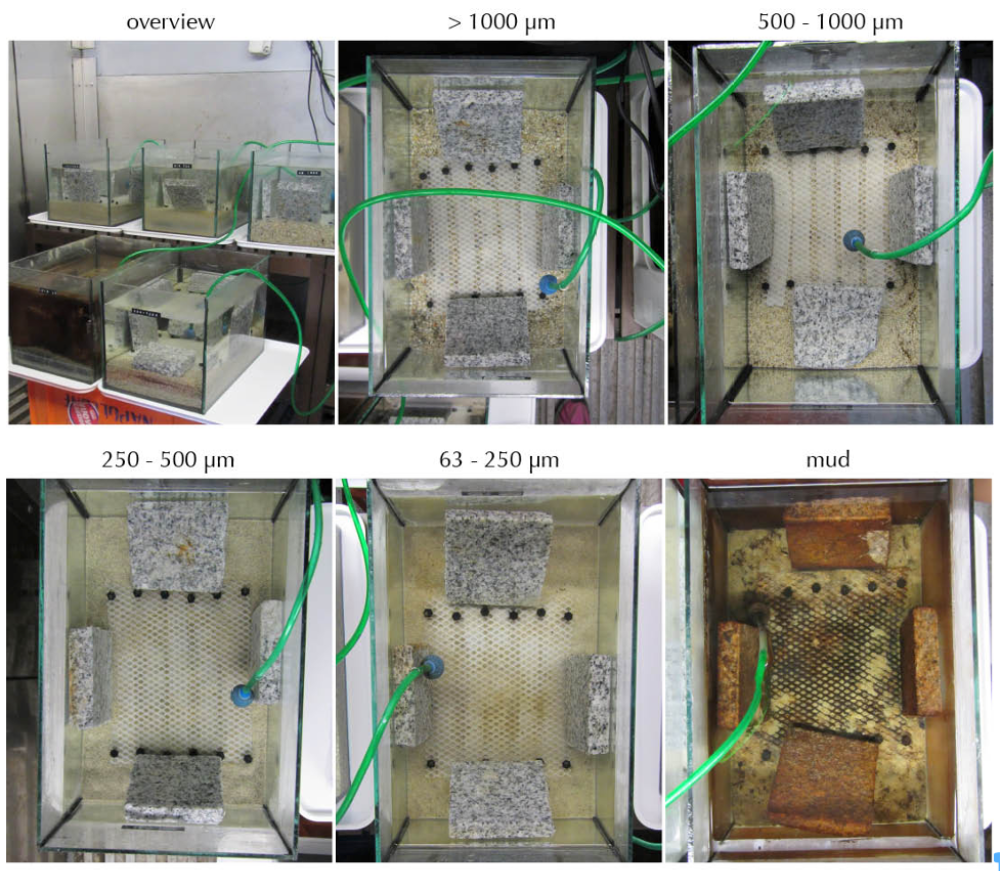

Supplement: Supplemental Information 2 — Overview and individual tanks with sand of the grain size fractions of >1,000 µm, 500–1,000 µm, 250–500 µm, 63–250 µm, and mud. Sample strips of Mater-Bi HF03V were placed between two layers of mesh and directly exposed at the sediment surface. Each mesh was weighted with 4 pieces of granite to secure it on the sediment surface. [file peerj-09-11981-s002.png]

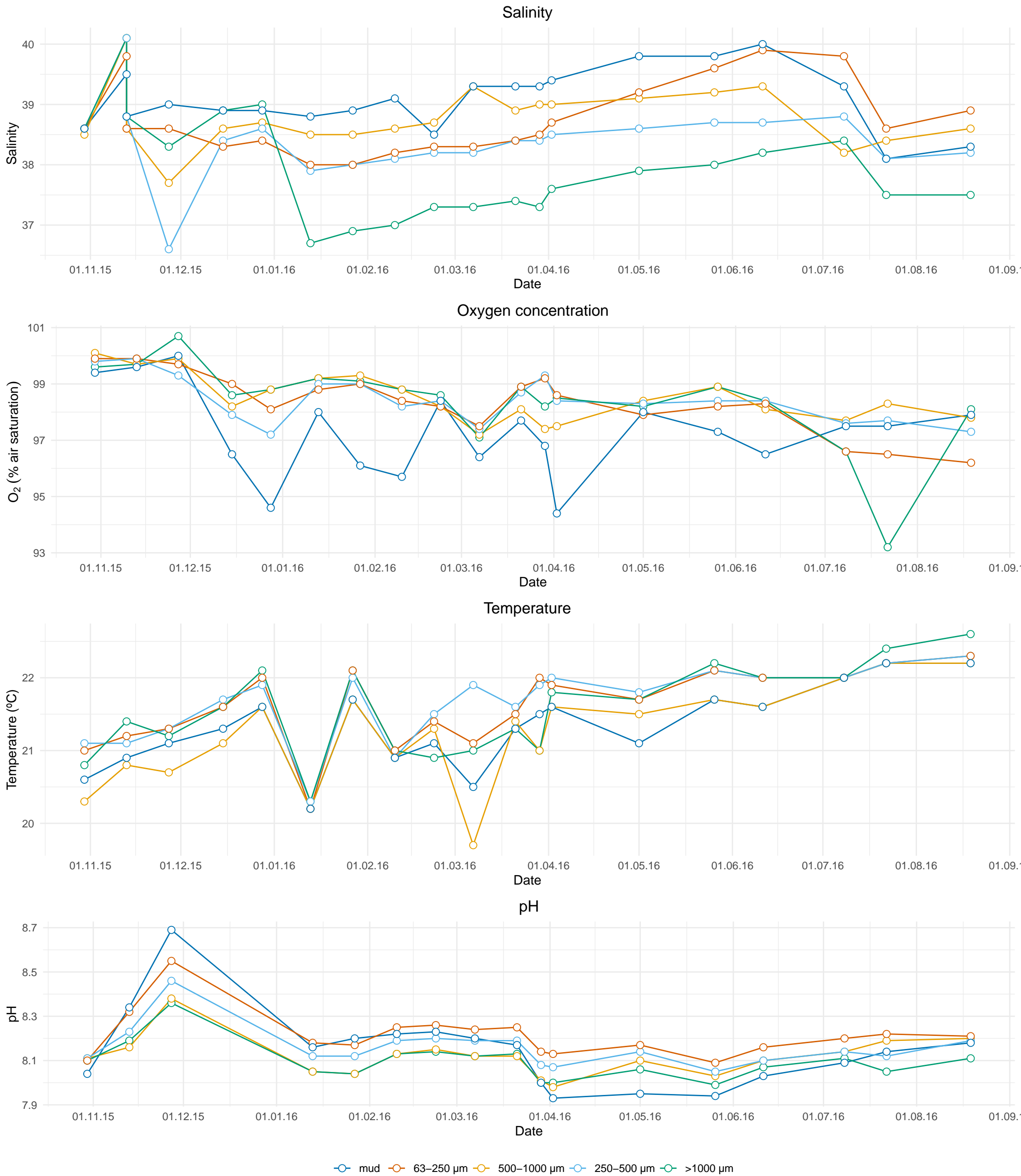

Supplement: Supplemental Information 3 [file peerj-09-11981-s003.pdf]

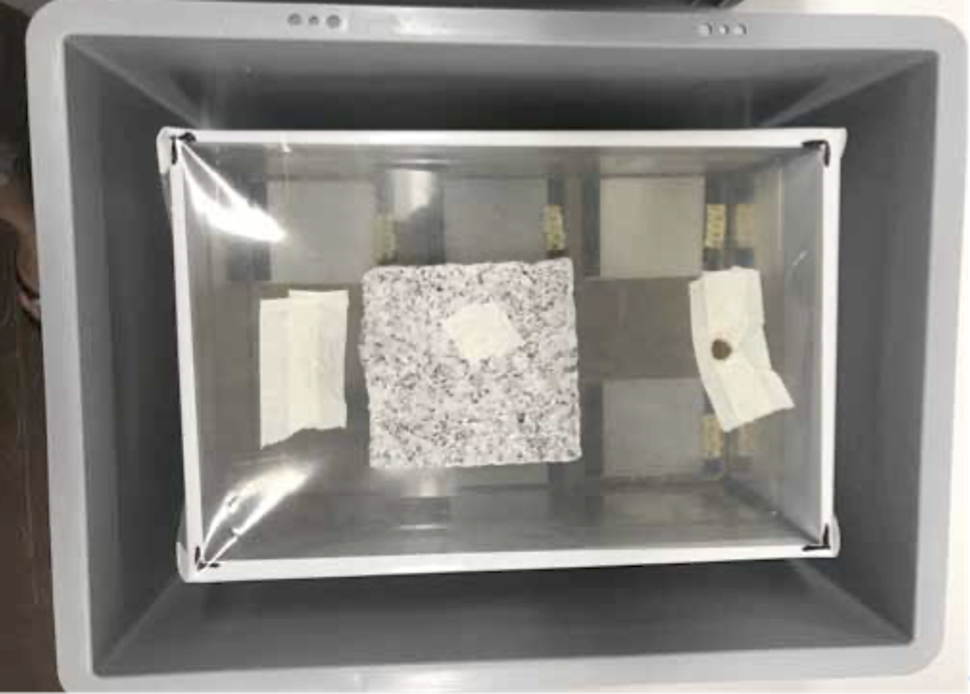

Supplement: Supplemental Information 4 — The sample film was protected by two layers of mesh and placed on the surface of sediment collected at different beaches. The lid covering the tank was weighted down with a granite tile. A hose connected to an air pump kept the oxygen concentration in the seawater at air-saturation. The tanks were placed in plastic bins. [file peerj-09-11981-s004.png]

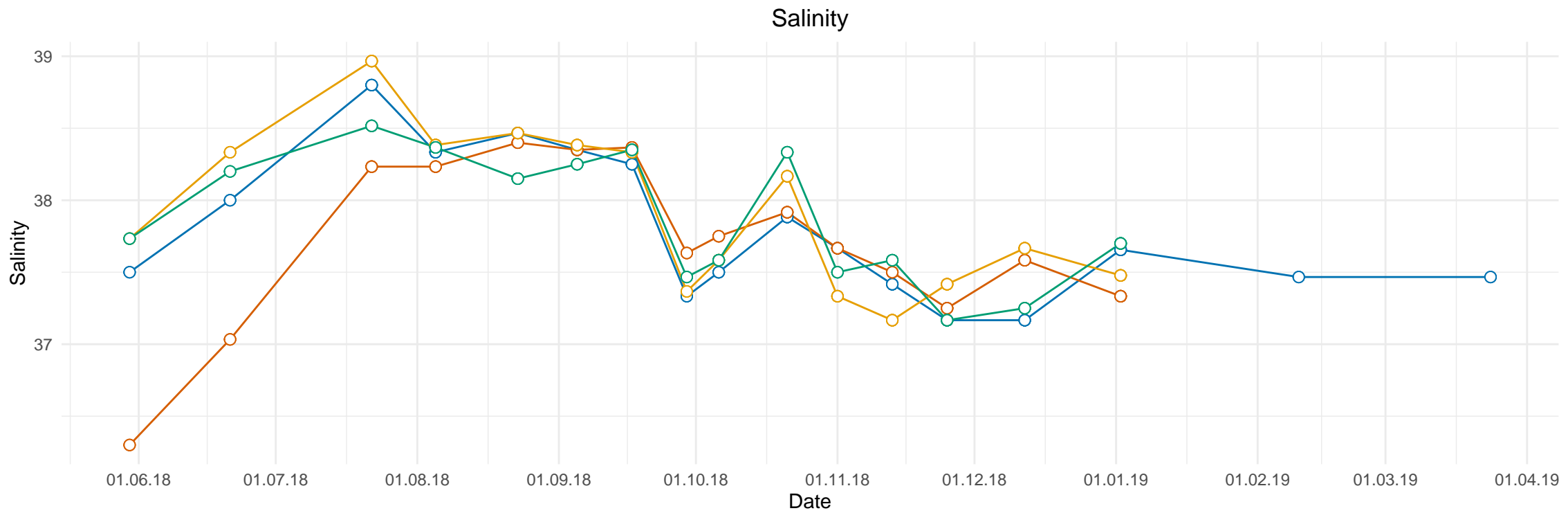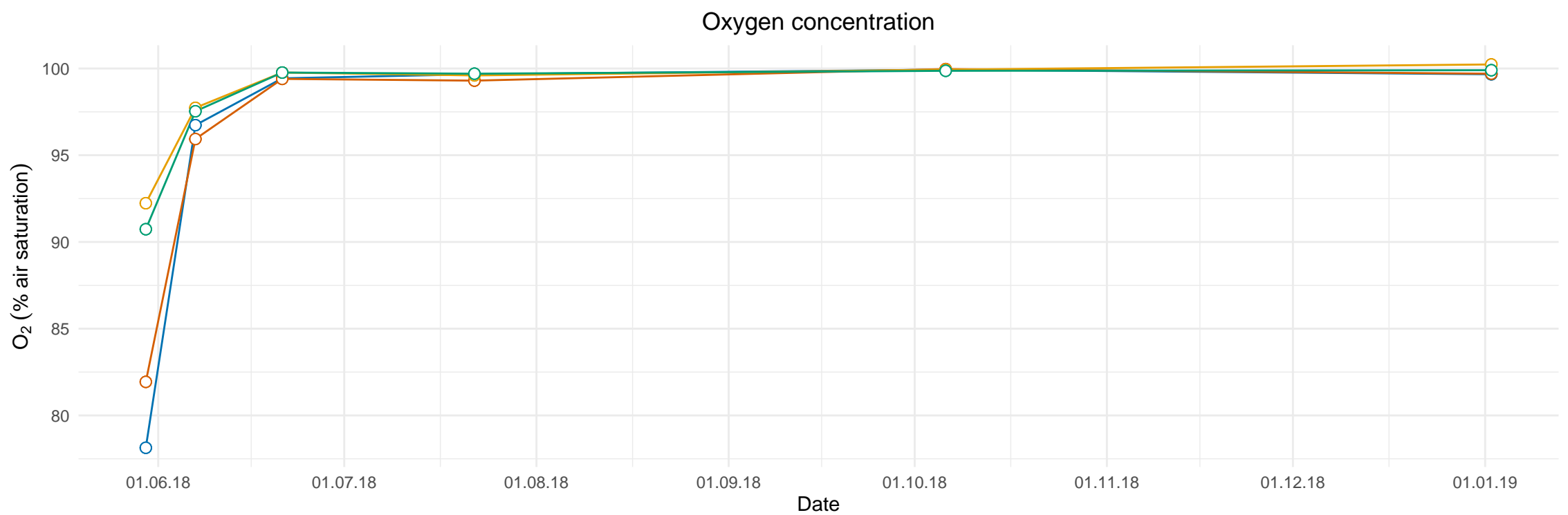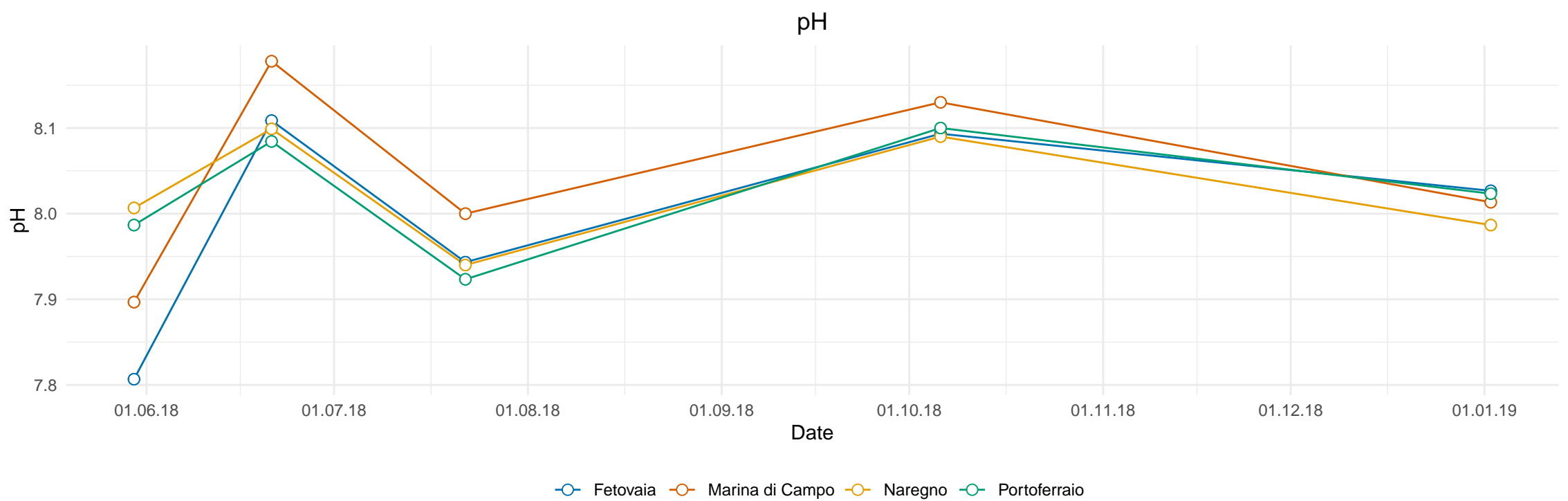

Supplement: Supplemental Information 5 [file peerj-09-11981-s005.pdf]

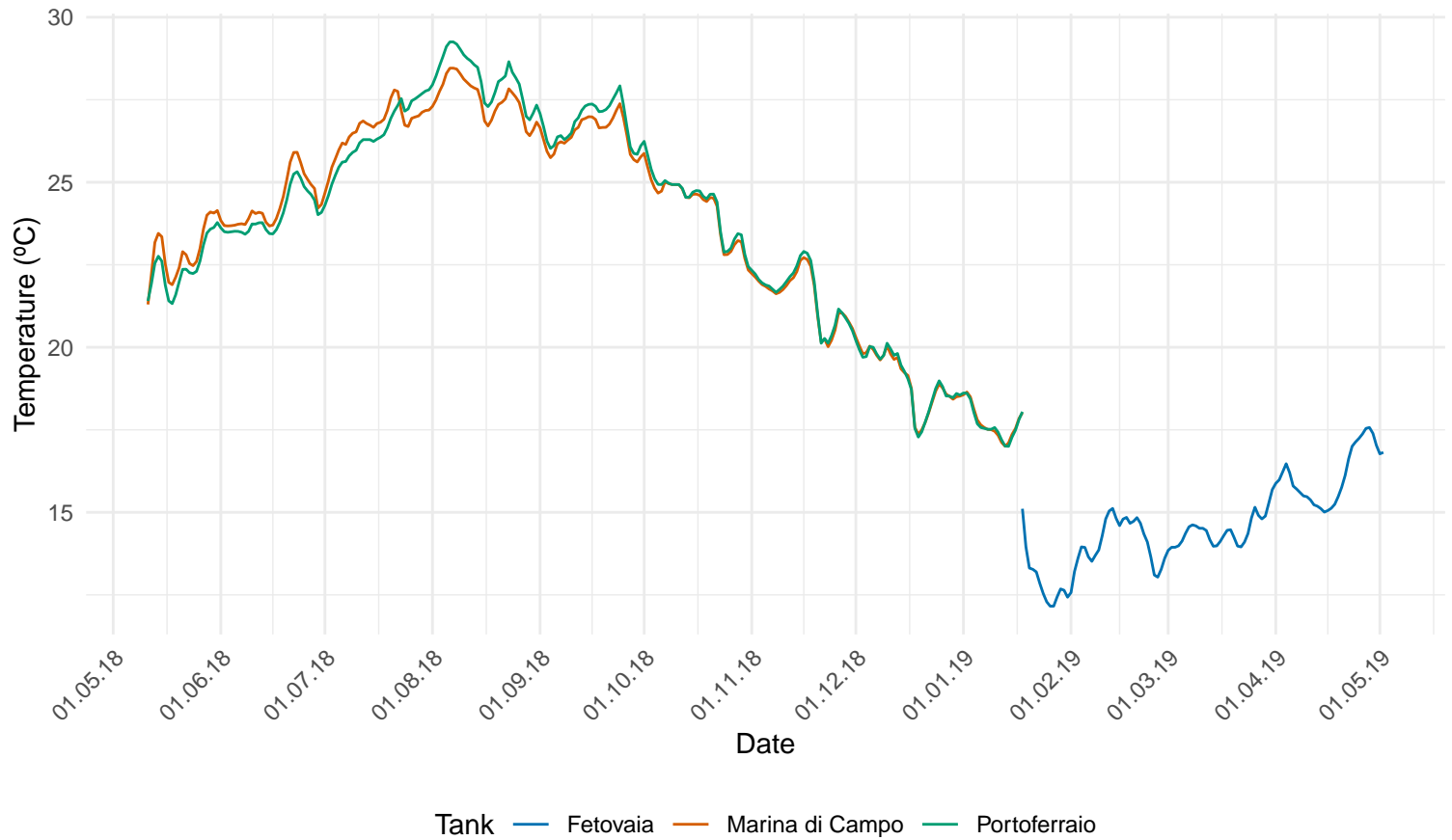

Supplement: Supplemental Information 6 — During the first phase of the experiment, the tanks containing Marina di Campo and Portoferraio sediment were monitored, the same temperatures are assumed for the tanks containing Fetovaia and Naregno sediment. Tanks containing Fetovaia sediment were monitored in the last phase of the experiments after the other tests were terminated. [file peerj-09-11981-s006.pdf]
