# Supplementary material for: Disintegration half-life of biodegradable plastic films on different marine beach sediments
Source: PeerJ. 2021 Aug 10;9:e11981. doi: 10.7717/peerj.11981 (PMC8362673; doi:10.7717/peerj.11981)

A)

Performance of *Mater-Bi* on all 4 sediments after 4 months

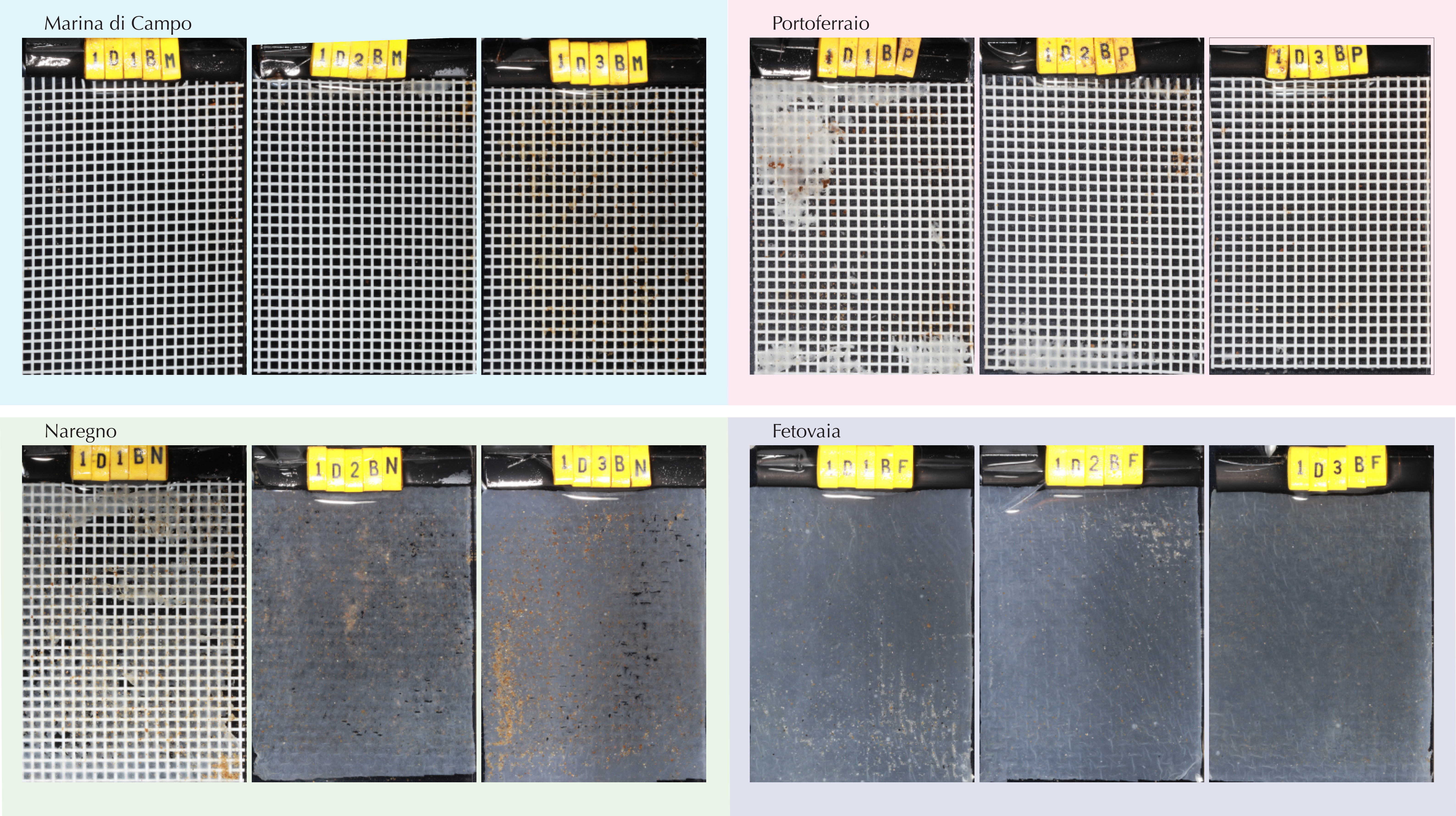

B)

Performance of *Mater-Bi* on all 4 sediments after 6 months

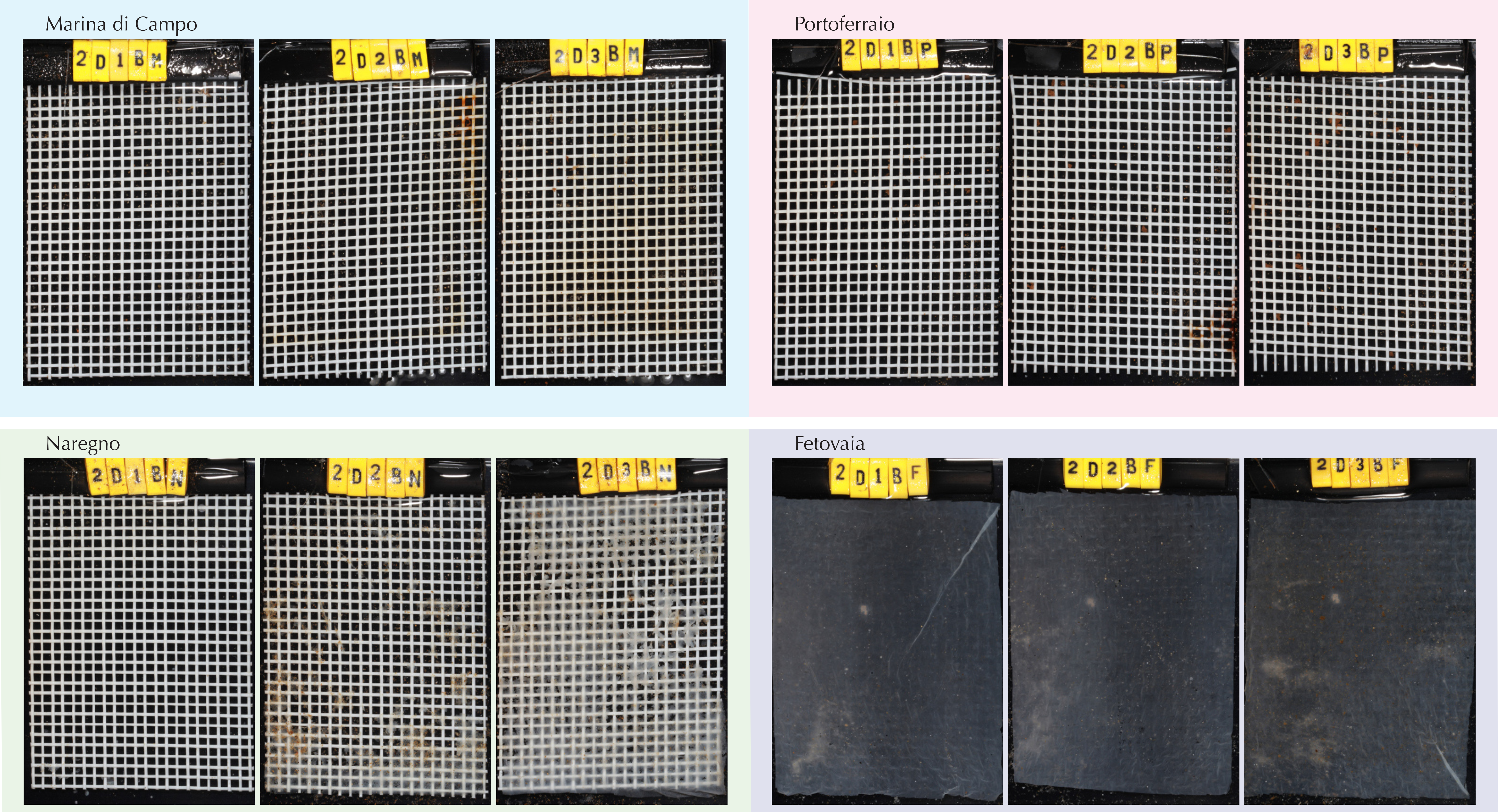

*Specimen 2D2BN adhering to both meshes.
See pages 36–37for details.

C)

Performance of *Mater-Bi* on 3 sediments after 8 months, and on 1 sediment after 12 months

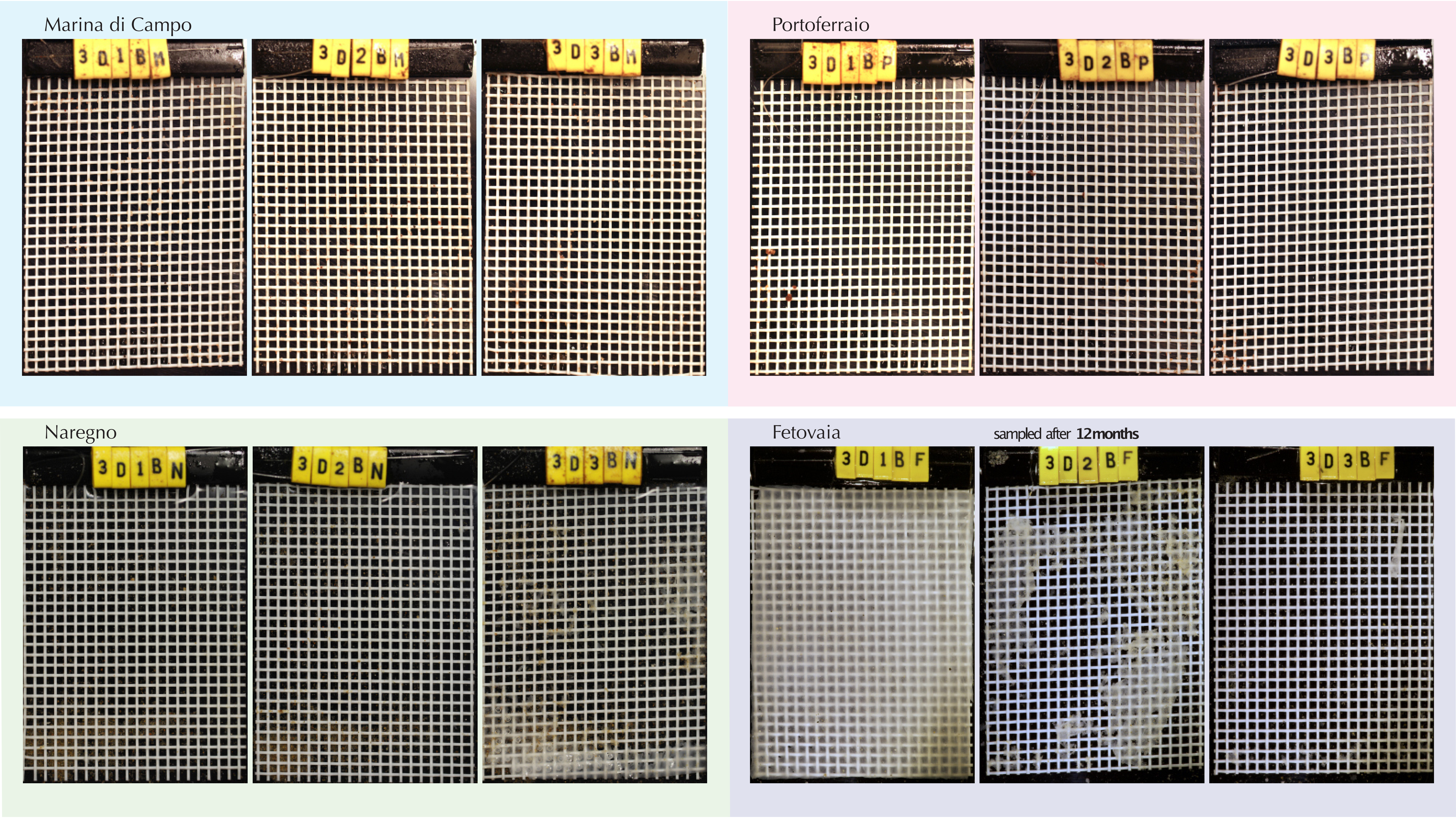

D)

Performance of *PHB 85 µm* on all 4 sediments after 4 months

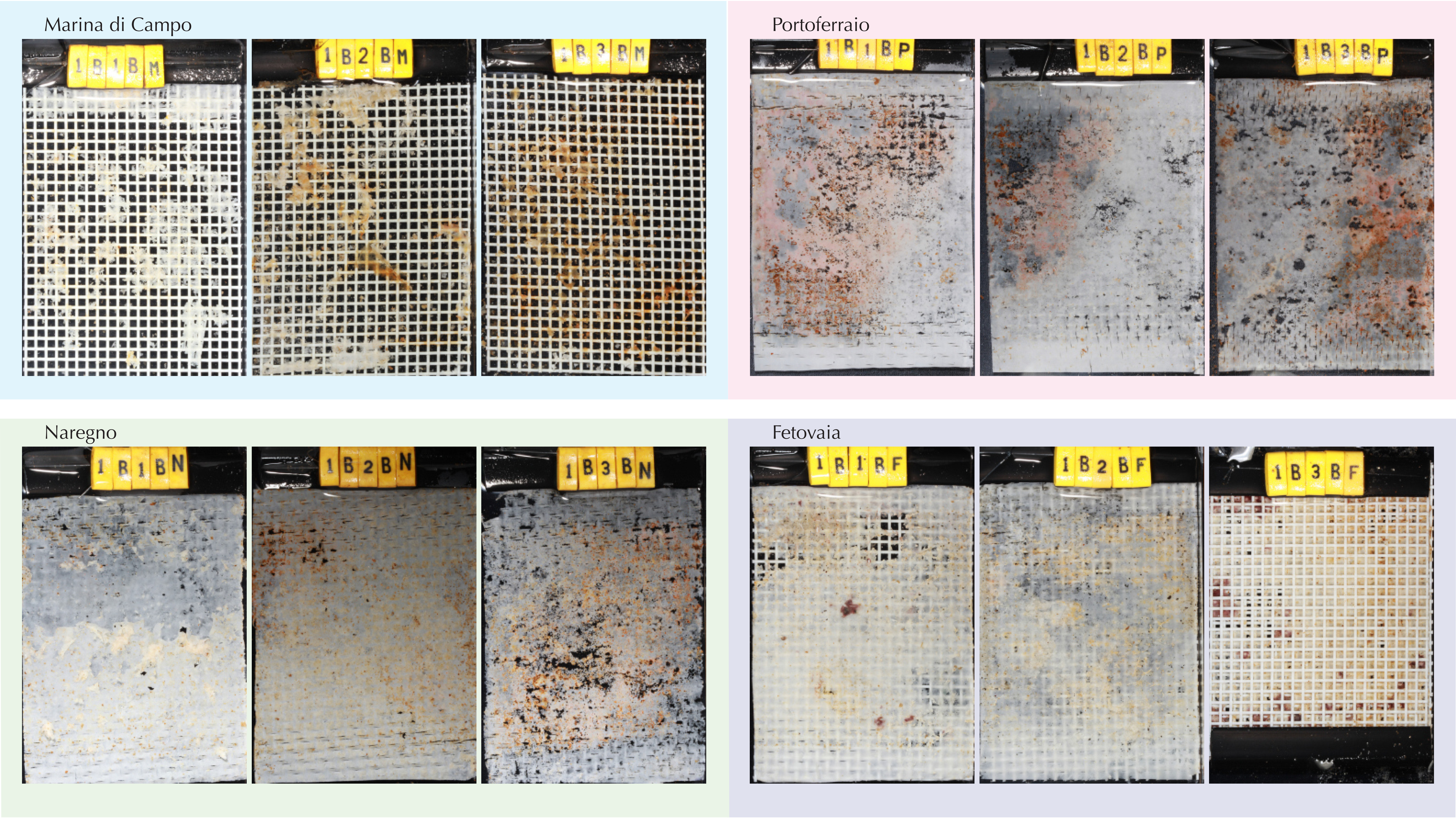

E)

Performance of *PHB 85 μm* on all 4 sediments after 6 months

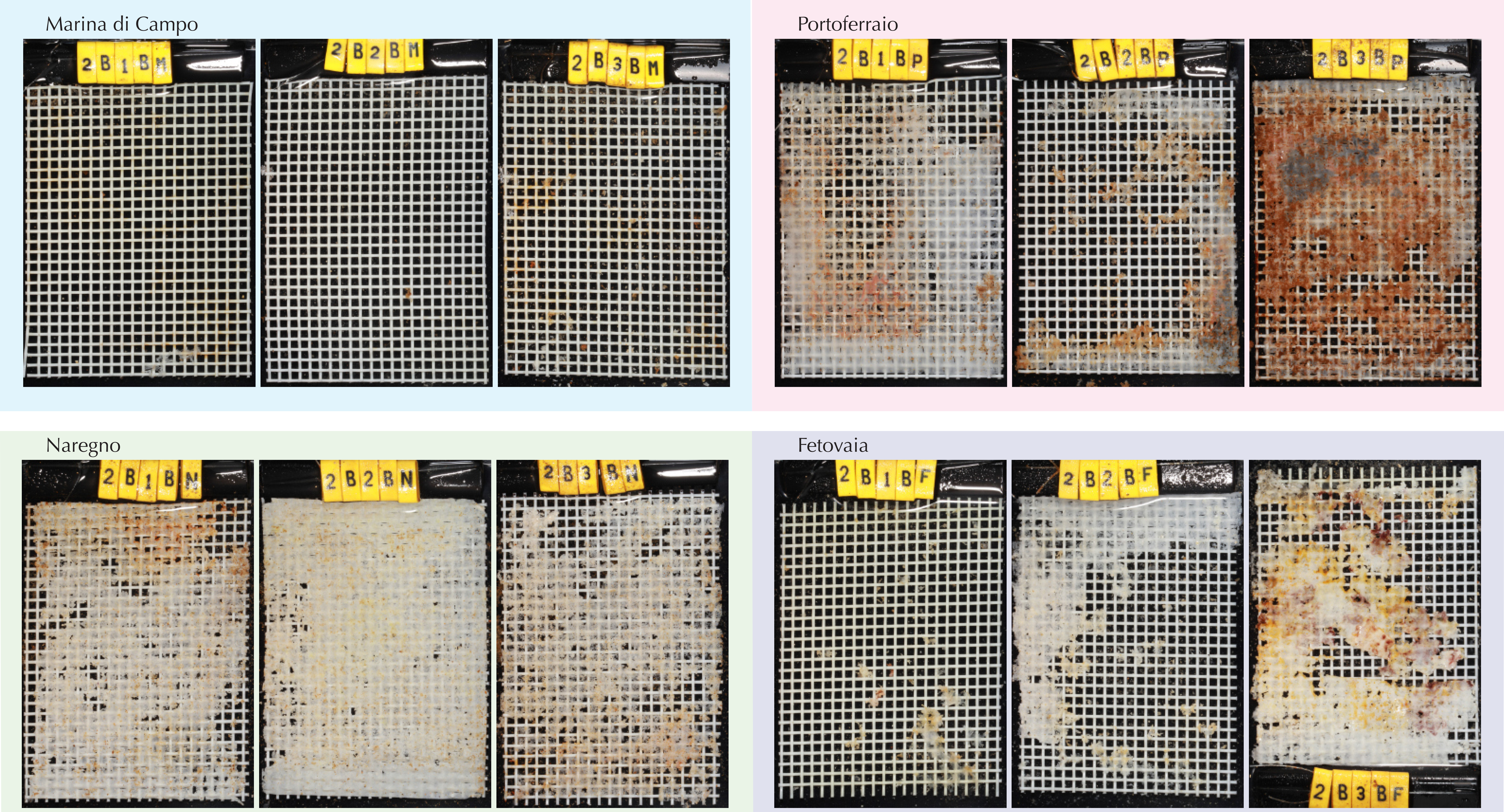

*Specimen 2B2BF adhering to both meshes.
See pages 54–55for details.

F)

Performance of *PHB 85 μm* on 3 sediments after 8 months, and on 1 sediment after 12 months

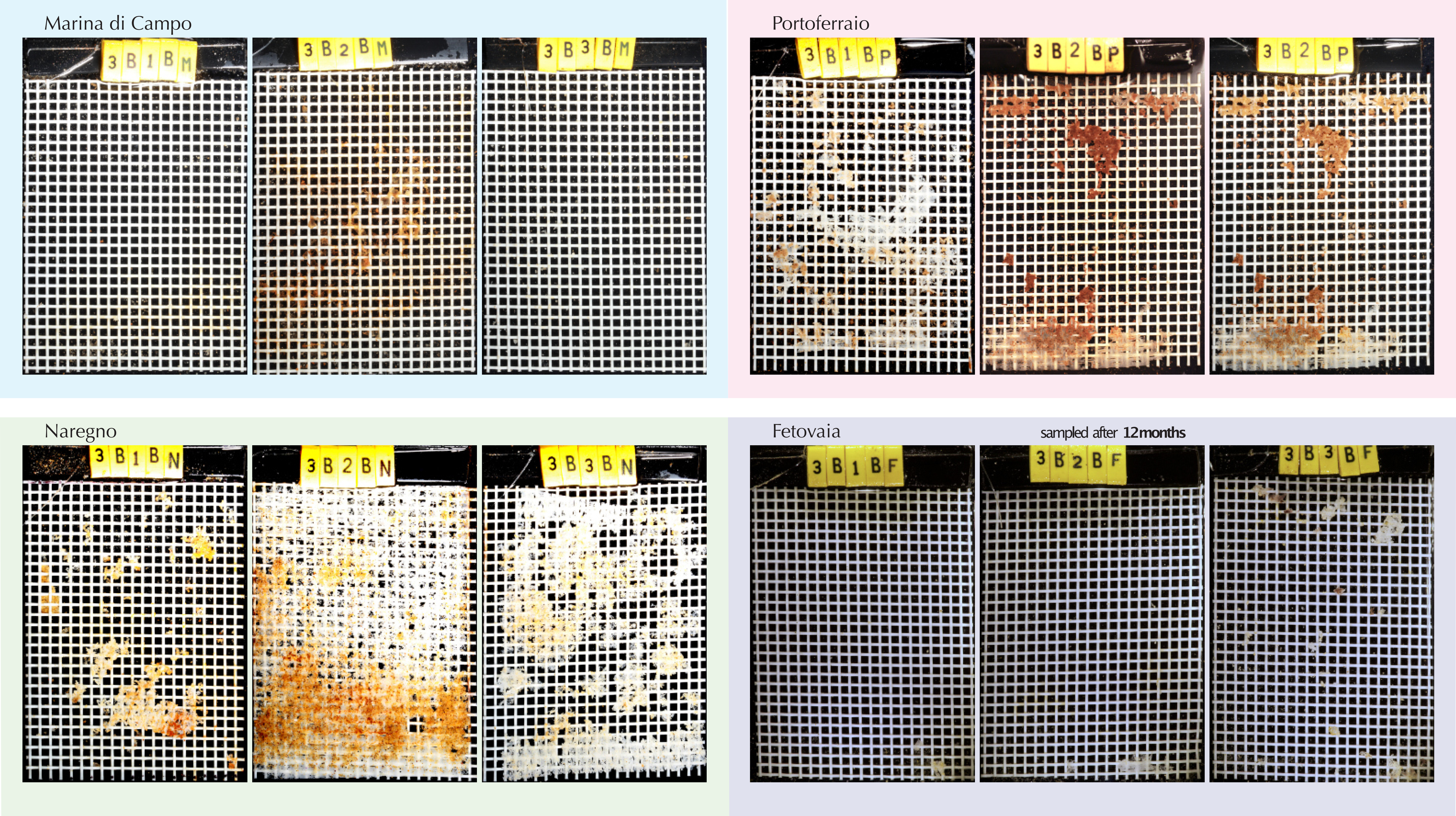

Supplement: Supplemental Information 7 — Depending on the state of material disintegration, the sample films were either photographed without the lower mesh or on the lower mesh. [file peerj-09-11981-s007.pdf]
