# Supplementary material for: Disintegration half-life of biodegradable plastic films on different marine beach sediments
Source: PeerJ. 2021 Aug 10;9:e11981. doi: 10.7717/peerj.11981 (PMC8362673; doi:10.7717/peerj.11981)
Supplement: Supplemental Information 8 — CL = 95% confidence level. [file peerj-09-11981-s008.docx]

| **Grain size fraction** | **t_0.5_** | **Lower CL** | **Upper CL** |
| --- | --- | --- | --- |
| mud | 139.4 | 134.1 | 143.1 |
| 63-250 µm | 296.3 | 293.9 | 298.6 |
| 250-500 µm | 309.8 | 306.9 | 313.8 |
| 500-1000 µm | 438.2 | 398.6 | 505.9 |
| >1000 µm | 428.1 | 385.8 | 509.2 |
