# Supplementary material for: Disintegration half-life of biodegradable plastic films on different marine beach sediments
Source: PeerJ. 2021 Aug 10;9:e11981. doi: 10.7717/peerj.11981 (PMC8362673; doi:10.7717/peerj.11981)
Supplement: Supplemental Information 9 — Significant p-values in bold. [file peerj-09-11981-s009.docx]

| **Group 1** | **Group 2** | ***p*-value** |
| --- | --- | --- |
| >1000 µm | 250-500 µm | **<0.0001** |
| >1000 µm | 500-1000 µm | 0.7928 |
| >1000 µm | 63-250 µm | **<0.0001** |
| >1000 µm | mud | **<0.0001** |
| 250-500 µm | 500-1000 µm | **<0.0001** |
| 250-500 µm | 63-250 µm | **<0.0001** |
| 250-500 µm | mud | **<0.0001** |
| 500-1000 µm | 63-250 µm | **<0.0001** |
| 500-1000 µm | mud | **<0.0001** |
| 63-250 µm | mud | **<0.0001** |
