# Supplementary material for: Disintegration half-life of biodegradable plastic films on different marine beach sediments
Source: PeerJ. 2021 Aug 10;9:e11981. doi: 10.7717/peerj.11981 (PMC8362673; doi:10.7717/peerj.11981)
Supplement: Supplemental Information 10 — One sample of bulk seawater before the experiments was analysed. TC, total carbon; TOC, total organic carbon; DOC, dissolved organic carbon; TIC, total inorganic carbon; POC, particulate organic carbon; DOP, dissolver organic carbon; TIN, total inorganic nitrogen; TON, total organic nitrogen; n.d., not detectable, i.e. below detection limit. [file peerj-09-11981-s010.docx]

| **Parameter** | **Unit** | **Value** |
| --- | --- | --- |
| TC | mg L^-1^ | 33 |
| TOC | mg L^-1^ | 1.6 |
| Nitrogen (Total-N) | mg L^-1^ | <0.25 |
| Phosphorus (Total-P) | mg L^-1^ | 0.12 |
| Ortho-phosphate-phosphorus (PO_4_-P) | mg L^-1^ | 0.008 |
| Phosphorus (Total-P) | mg L^-1^ | 0.065 |
| DOC | mg L^-1^ | 1.6 |
| Nitrate-nitrogen (NO_3_-N) | mg L^-1^ | 0.14 |
| Nitrite-nitrogen (NO_2_-N) | mg L^-1^ | <0.02 |
| Ammonium-nitrogen (NH_4_-N) | mg L^-1^ | 0.13 |
| TIC | mg L^-1^ | 31.4 |
| POC | mg L^-1^ | 0 |
| DOP | mg L^-1^ | 0.065 |
| TIN | mg L^-1^ | 0.27 |
| TON | mg L^-1^ | n.d. |
