# Supplementary material for: Disintegration half-life of biodegradable plastic films on different marine beach sediments
Source: PeerJ. 2021 Aug 10;9:e11981. doi: 10.7717/peerj.11981 (PMC8362673; doi:10.7717/peerj.11981)
Supplement: Supplemental Information 11 — CL = 95% confidence level [file peerj-09-11981-s011.docx]

| **Beach** | **Material** | **t_0.5_** | **Lower CL** | **Upper CL** |
| --- | --- | --- | --- | --- |
| Fetovaia | Mater-Bi HF03V | 368.0 | 243.4 | 677.0 |
| Marina di Campo | Mater-Bi HF03V | 72.3 | 55.1 | 86.9 |
| Naregno | Mater-Bi HF03V | 176.7 | 142.4 | 207.6 |
| Portoferraio | Mater-Bi HF03V | 90.7 | 62.6 | 107.3 |
| Fetovaia | PHB | 184.4 | 163.2 | 212.3 |
| Marina di Campo | PHB | 111.7 | 97.1 | 119.8 |
| Naregno | PHB | 259.3 | 223.0 | 361.9 |
| Portoferraio | PHB | 215.0 | 197.0 | 236.7 |
