# Supplementary material for: Disintegration half-life of biodegradable plastic films on different marine beach sediments
Source: PeerJ. 2021 Aug 10;9:e11981. doi: 10.7717/peerj.11981 (PMC8362673; doi:10.7717/peerj.11981)
Supplement: Supplemental Information 12 — Significant p-values in bold. [file peerj-09-11981-s012.docx]

| **Group 1** | **Group 2** | ***p*-value** |
| --- | --- | --- |
| Fetovaia | Marina di Campo | 0.1056 |
| Fetovaia | Naregno | 0.1056 |
| Fetovaia | Portoferraio | 0.1056 |
| Marina di Campo | Naregno | **0.0393** |
| Marina di Campo | Portoferraio | 0.2067 |
| Naregno | Portoferraio | **0.0436** |
